# Human-centered design of clinical decision support for management of hypertension with chronic kidney disease

**DOI:** 10.1186/s12911-022-01962-y

**Published:** 2022-08-13

**Authors:** Pamela M. Garabedian, Michael P. Gannon, Skye Aaron, Edward Wu, Zoe Burns, Lipika Samal

**Affiliations:** 1 Mass General Brigham, 399 Revolution Drive, Somerville, MA 857-282-4091 USA; 2 Division of General Internal Medicine, Brigham and Women’s Hospital, Boston, MA USA; 3 Alabama College of Osteopathic Medicine, Dothan, AL USA; 4 Harvard Medical School, Boston, MA USA

**Keywords:** Human-centered design, Clinical decision support systems, Health information technology

## Abstract

**Background:**

Primary care providers face challenges in recognizing and controlling hypertension in patients with chronic kidney disease (CKD). Clinical decision support (CDS) has the potential to aid clinicians in identifying patients who could benefit from medication changes. This study designed an alert to control hypertension in CKD patients using an iterative human-centered design process.

**Methods:**

In this study, we present a human-centered design process employing multiple methods for gathering user requirements and feedback on design and usability. Initially, we conducted contextual inquiry sessions to gather user requirements for the CDS. This was followed by group design sessions and one-on-one formative think-aloud sessions to validate requirements, obtain feedback on the design and layout, uncover usability issues, and validate changes.

**Results:**

This study included 20 participants. The contextual inquiry produced 10 user requirements which influenced the initial alert design. The group design sessions revealed issues related to several themes, including recommendations and clinical content that did not match providers' expectations and extraneous information on the alerts that did not provide value. Findings from the individual think-aloud sessions revealed that participants disagreed with some recommended clinical actions, requested additional information, and had concerns about the placement in their workflow. Following each step, iterative changes were made to the alert content and design.

**Discussion:**

This study showed that participation from users throughout the design process can lead to a better understanding of user requirements and optimal design, even within the constraints of an EHR alerting system. While raising awareness of design needs, it also revealed concerns related to workflow, understandability, and relevance.

**Conclusion:**

The human-centered design framework using multiple methods for CDS development informed the creation of an alert to assist in the treatment and recognition of hypertension in patients with CKD.

**Supplementary Information:**

The online version contains supplementary material available at 10.1186/s12911-022-01962-y.

## Background

There is a need to improve recognition and control of hypertension in patients with chronic kidney disease (CKD). Among patients with CKD, 52% have diagnosed hypertension, 19% have pre-hypertension, and 16% have undiagnosed hypertension [1]. Of the patients with CKD and uncontrolled hypertension, just 40% are prescribed anti-hypertensive medications [2]. Additionally, less than 10% of patients with an estimated glomerular filtration rate (eGFR) under 60 mL/min/1.73 m^2^ are aware that they have CKD, and just 15% of patients with CKD had a documented diagnosis [2]. One method to assist physicians in the recognition and management of these patients is to provide decision support for the primary care provider within the electronic health record.

Clinical decision support (CDS) provides “clinicians or patients with clinical knowledge and patient-related information, intelligently filtered or presented at appropriate times, to enhance patient care.” [3] While rapid adoption of electronic health records (EHR) has led to increased CDS use, some studies suggest that primary care providers (PCPs) are resistant to its implementation [4–7]. In practice, CDS can lead to unintended consequences like alert fatigue, workflow obstruction, increased workload, and alert dismissal [8–10]. These issues are more prevalent when the CDS fits workflow poorly, has a low alert severity level, lacks informational sources, has a poor layout, fires multiple times per encounter, fires on multiple patients a day, or does not match providers’ mental models of disease [11–16]. Given that the success of novel CDS depends on whether providers use it, development and design should include feedback and input from intended users to create a usable system [17]. This feedback should aim to improve understanding of clinical tasks, workflows, physician use of the EHR, and organizational culture [18–20].

Previous goals of CDS design have centered around efficiency, error rates, and guideline adherence rather than the overall usability of CDS features [21, 22]. While the CDS may be efficient, there is a greater possibility of adverse clinical outcomes when formal requirements gathering and usability testing has not focused on user preferences. These outcomes include inappropriate prescriptions, under- and overprescribing, medical errors, and CDS dismissal [23–28]. While many studies have addressed the layout, timing and efficiency, or informativeness of CDS, usability issues may still persist [29].

In this study, we followed a human-centered design (HCD) process that solicits feedback from clinicians at multiple stages and using multiple methods. Human-centered design can be defined as “an approach to interactive systems that aims to make systems usable and useful by focusing on the users, their needs and requirements, and by applying human factors/ergonomics, and usability knowledge and techniques. This approach enhances effectiveness and efficiency, improves human well-being, user satisfaction, accessibility and sustainability; and counteracts possible adverse effects of use on human health, safety and performance.” [30] Human-centered design has become a more common design and development process for provider and patient facing health IT applications in the last 10 years [31–35]. Following this approach has been shown to result in systems that are easier to use and lead to greater adoption [36–39].

The use of CDS has promise in the context of CKD, with recent studies showing that it leads to an increase in the rate of urine albumin monitoring [23, 40]. However, there was not a significant decrease in blood pressure (BP) in prior studies of CDS for CKD [40]. These studies primarily focused on CKD, alerting clinicians to order referrals to nephrology or ordering additional urine tests. Our study aimed to develop and validate CDS that synthesized existing EHR data (laboratory tests, medication orders, and vital signs) to increase recognition of CKD and uncontrolled hypertension in CKD patients and deliver evidence-based personalized CKD and hypertension management recommendations. Specifically, we designed alerts for three overarching categories of CDS that recommend (1) initiation of a specific class of anti-hypertension medication [Angiotensin Converting Enzyme Inhibitor (ACE)/Angiotensin Receptor Blocker (ARB)], (2) increasing the dose of ACE/ARB medications, and (3) initiation of a diuretic (Hydrochlorothiazide) in patients already prescribed the maximum dose of an ACE/ARB. The objective of the study was to improve the CDS content and design using an iterative human-centered design strategy. Through this process, we aimed to create CDS that fits the providers’ workflow, presents relevant data and recommendations, and promotes higher quality care for patients with CKD.

## Methods

**Fig. 1:**
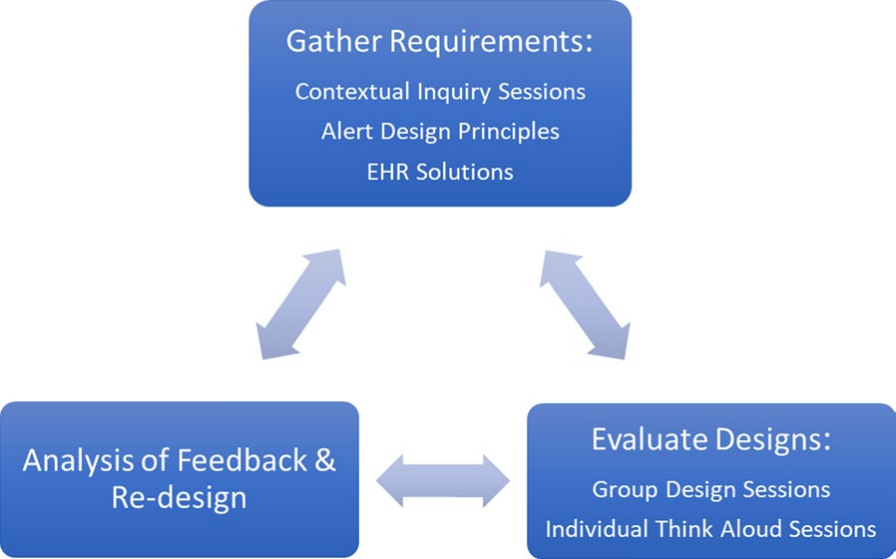
Human-centered design process for design of a best practice advisory

To design and develop the alerts, we followed a human-centered design process focusing on the involvement of users at each stage of design and development, an understanding of user needs and an iterative process (Fig. 1). Prior to creating prototypes of the alerts, we conducted contextual inquiry sessions to help gather user requirements for the alerts. Group design sessions along with iterative design were used to validate existing requirements and gather additional requirements and feedback on alert design and layout. Finally, we conducted one-on-one formative usability sessions to uncover additional issues and evaluate existing design decisions. All sessions were recorded using Morae (TechSmith Corporation, Okemos, Michigan) screen recording software and a backup digital audio recorder. This study was approved by the Mass General Brigham Institutional Review Board under the Human Research Protection Program.

### Recruitment

Primary Care Physicians (PCPs) from Brigham and Women’s 15 affiliated primary care clinics were emailed a recruitment letter and frequently asked questions document by the principal investigator. Based on the simplicity of the system interface and the iterative process, the recruitment goal was a minimum of 18 total participants with 6–8 per activity until we reached saturation in our findings [41, 42]. A research team member followed up to schedule sessions with interested participants. Participants who responded after we reached our expected number of participants for that activity were invited to participate in subsequent activities. Informed consent was obtained from all participants.

### Alert design

Brigham and Women’s affiliated primary care clinics use Epic Hyperspace (Epic Systems Corporation, Verona, Wisconsin) as the EHR. Best Practice Advisories (BPAs) were used as the format of CDS for alerting providers and giving treatment recommendations. Our decision support was intended to address three categories of antihypertensive treatment in CKD: initiation of ACE/ARB treatment, increasing dose, and adding a diuretic [43–46]. Providers have the option of accepting the alert or overriding it. Acknowledge reasons allow the provider to give a reason for overriding the alert, either from a coded list or a free-text description [47]. Further description of the CDS is described in other publications [46, 48].

### Contextual inquiry sessions

We gathered user requirements through contextual inquiry sessions. The goal of the inquiry sessions was to understand the different activities, steps, and thinking processes involved in managing uncontrolled blood pressure using the EHR, to generate user requirements for CDS.

The contextual inquiry sessions were conducted at the participant’s office or virtually, using their own computer with the software that they use to do their daily work. The moderator provided the participant with a short introduction that included information about the structure of the session and demographic questions. Participants were interviewed on their use of the EHR to manage chronic disease, the overall structure of their visits, and any challenges or issues important to them in regard to hypertension. During the second part of the session, participants were asked to show and explain their process of how they prepare for and then go through an encounter with a patient with CKD and uncontrolled hypertension, their workflow and how they use the EHR to support their activities. Clinical scenarios were used to understand how the providers interact with the EHR to support their decision making related to the patient’s management of CKD and hypertension.

### Group design sessions

**Fig. 2:**
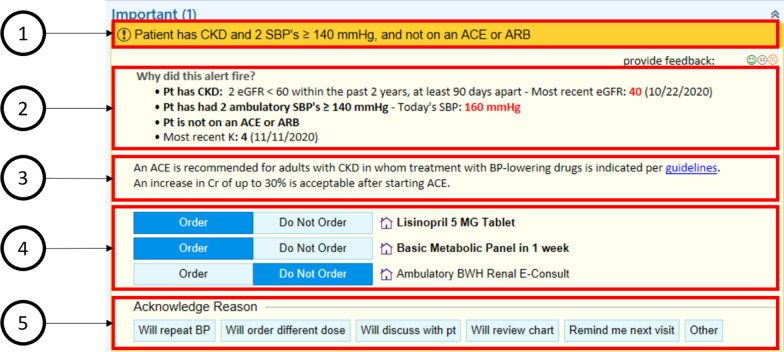
Alert structure. 1: justification; 2: relevant data; 3: guidelines/additional information; 4: order options; 5: acknowledge reasons

Based on the user requirements elucidated in the contextual inquiry sessions, we designed several mockups of the alerts in categories 1 and 2 (initiation of an anti-hypertensive medication and increased dose of an anti-hypertensive medication, respectively). Not all user requirements could be incorporated due to limitations of the EHR. The structure of each mock-up included the rationale and relevant statistics, guidelines, order options and acknowledge reasons (Fig. 2). To validate user requirements and learn more about provider preferences, we presented mockups to focus group participants, with several options for each category of alert. Participants were asked open-ended questions, such as “What is the first thing you notice?”, “Tell us what you like about what you are seeing here”, and “Tell us what you don’t like.” Due to the COVID-19 pandemic, group design sessions were conducted via videoconferencing using Zoom (Zoom Video Communications, San Jose, California).

### Individual think aloud sessions

Based on the results of the design sessions, we refined the alert design. We also added an additional mockup for a third category of alerts (initiation of a diuretic (HCTZ) in patients already prescribed the maximum dose of an ACE/ARB). We developed working versions of the alerts that would trigger in the sandbox environment of the EHR to use during the next phase of usability testing.

We conducted individual formative think aloud usability testing sessions to uncover additional usability issues and validate design decisions. We developed five scenarios in which one of the five alert variations would trigger (Additional file 1: Appendix A). A research team member logged into the environment and passed control of the mouse and keyboard to the participant so that they could interact with the EHR. We made changes to the mockups after conducting sessions with the first four participants and tested the newer versions with the remaining four participants.

### Analysis

The human factors specialist (PG) reviewed the recordings of the contextual inquiry sessions to identify common themes across participants. The themes were reviewed with the broader research team. Themes were then translated into user requirements. Potential solutions to address these requirements in the CDS were brainstormed and subsequently investigated for feasibility within the EHR by the research team.

Group design sessions were transcribed. Transcriptions were reviewed by two members of the research team (SA, EW) who independently coded participant comments by feature/functionality and design element and came together with the human factors specialist to review discrepancies, revise codes, and reach consensus. The human factors specialist (PG) reviewed the coded comments and grouped them into 7 major themes.

Two research team members (SA, EW) reviewed the transcripts and video of the usability tests to identify usability issues encountered by the participants during the testing sessions. The research team members documented the individual user feedback, an associated quote, and contextual information describing the usability issue [49]. A content analysis was conducted by the research team to group individual participant usability issues and comments into a set of usability findings which were reviewed by the human factors specialist.

Throughout the design process, we held standing meetings with the research team and 1–2 additional subject matter experts in nephrology and informatics. Subject matter expert meetings were used to review user feedback, discuss requirements and make preliminary decisions about content and design. Build meetings were used to discuss the limitations of Epic and technical issues. All Team meetings included multiple highly experienced informatics researchers and clinical trial experts and was the final decision-making body.

## Results

Recruitment emails were sent to 212 PCPs. Of the respondents that indicated interest in participating in the human-centered design process, 6 took part in the contextual inquiry session, 9 in the group design sessions, and 8 in the individual think aloud sessions. 3 of the contextual inquiry participants also partook in the individual think aloud sessions for a total of 20 participants. 8 of the 20 participants were female and 12 were male. All were PCPs consisting of 18 physicians, 1 nurse practitioner and 1 physician assistant (Table 1).

**Table 1:** Participant characteristics and stage(s) of involvement

Activity	N	Gender		Role		
		Male	Female	MD	NP	PA
Contextual inquiry session	6	5	1	6	0	0
Group design session	9	5	4	8	1	0
Individual think aloud session	8	4	4	7	0	1

### Contextual inquiry sessions

The sessions were conducted from May 2019 to October 2019. Six participants agreed to participate. 5 out of the 6 participants were male. Participants averaged more than 17 years in clinical practice and have used the current EHR for 2–7 years.

The contextual inquiry sessions resulted in several insights related to how physicians use the EHR to structure their visits with patients, retrieve and document patient data specifically related to hypertension and CKD, and determine a plan for these patients.

We found that providers review labs and vitals with the patient in the exam room, as they work through each condition. Most providers preferred to view data graphically or in tabular format when available to identify trends and correlations between labs and medications. They also said that during the visit, they scan the data for abnormal values or other things that stand out. All providers used the note as the focal point of their visit by reviewing their previous notes and capturing important information in the current note. Providers also described typically talking with the patient first and addressing their reason for visit, then addressing other issues if time allowed.

Multiple providers described specific challenges faced when making decisions about adding or changing a medication for hypertension. Many challenges included issues with accessing relevant historical data such as medication history and side effects. In addition, knowledge of how adherent a patient is to their current medication regimens can be difficult to attain. Other providers highlighted the issues of capturing accurate BP values due to availability of appropriate cuffs or other factors affecting a patient’s BP. Providers often repeat BP measurements during the visit. In addition, home BP monitoring is captured only in provider notes and therefore can be difficult to track over time.

Multiple observations and comments from providers centered around accessing information about the context of a specific BP measurement. Information about where the BP was taken, when the BP was taken in relation to the time of the visit, how the BP was taken and the patient’s physical, emotional, and mental state when the BP was taken is often considered in the provider’s decision making.

Finally, the issue of alert fatigue was raised: many providers ignored alerts and expressed their concerns about receiving multiple alerts that often do not have clear and actionable messages. Providers expressed challenges in clearly identifying high priority alerts and how to interact with them.

The above insights were translated into 10 user requirements (Table 2) by the human factors specialist (PG).

**Table 2:** User requirements and potential solutions based on the insights from the contextual inquiry sessions

ID	User requirement	Potential solution(s)
1	The system shall provide the user with **information about the alert logic** and whether it has taken into consideration the medication reactions or allergies	Check for documented allergies/adverse reactions in the EHR and include information with the alert
2	The system shall provide the user with enough **context about the patient’s BP readings** so that the user can take context into consideration (e.g., falsely elevated BP due to pain)	Include the date the BP was taken, link to associated note, vital signs pain score Make it clear that the values are from the ambulatory BP flowsheet Acknowledge reasons should be based on BP history Explore existing problem-oriented views
3	User needs to know the **patients’ baseline BP** to interpret/make decisions	Show high BPs, and most recent BP
4	System should provide an easy way for the user to see **trends and correlations** between other patient data	Graph BP along with medications and labs, or vitals like weight Provide access to graphical views of trending data
5	User needs easier ways to **access BP measurements from home**, have patients take measurements at home	Integration with other hypertension management efforts across the health system
6	Discussion with patient about **medication adherence** needs to be part of the decision to change/add a medication; or have more information from pharmacy	Ideally, alerts would be suppressed if patient was non-adherent and we would provide separate warnings along the lines of “patient BP running high and not taking medications”
7	System provides user with important/relevant **information at the appropriate time during the visit** (when reviewing history of present illness)	Pop-up alert later in the visit after physician has repeated falsely elevated BP and has had time to talk with patient Explore available trigger points: opening chart, signing orders, closing chart
8	User can easily **identify values requiring attention** (deviating from patient’s baseline)	Prepopulate note with “outstanding labs and vitals,” and other things that need attention during the visit
9	System provides user with **guidelines and recommendations** that are integrated into note workflow/visit workflow	Alerts pull into note when related to a problem that is pulled in Prepopulate note with alert
10	System supports the user in **prioritizing patient reminders and alerts** and addressing them efficiently	Alerts should be patient specific, provider specific and context specific with one-click actionable recommendations Buttons to directly order medications from alert

### Group design sessions

**Fig. 3:**
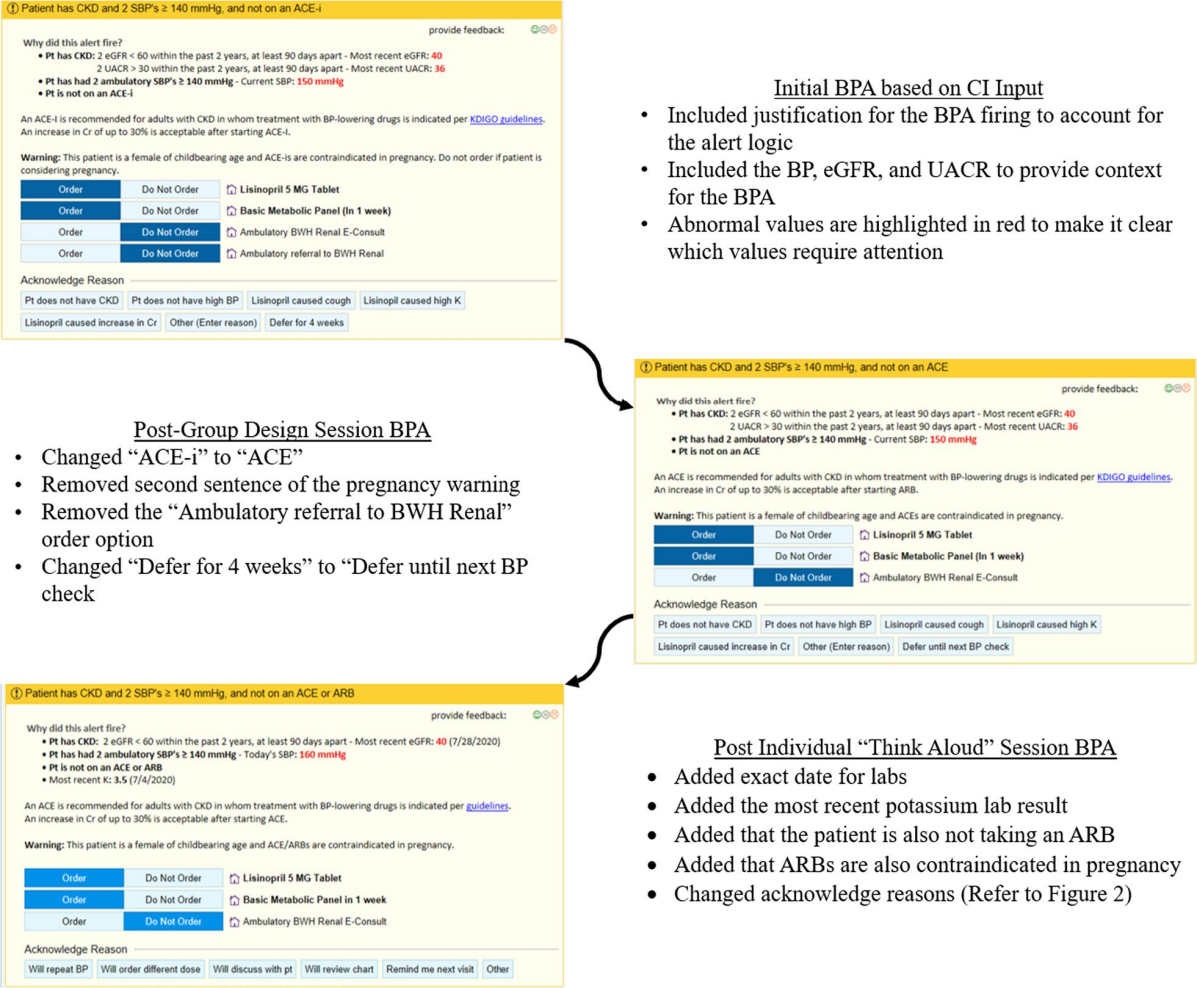
Changes in alert content and formatting through each iteration

**Fig. 4:**
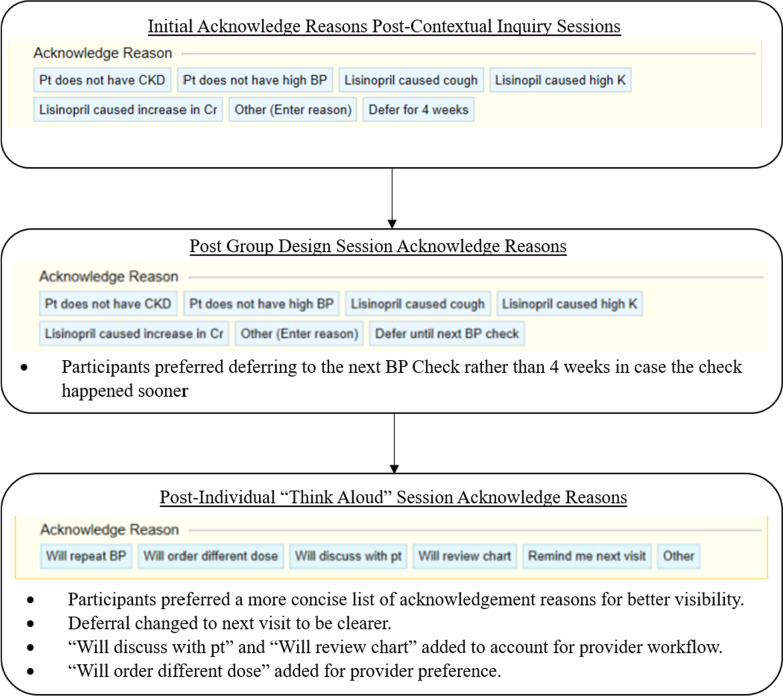
Specific changes to the acknowledge reasons. These were made to better fit provider needs and workflow

These sessions were held in April 2020. We recruited 9 participants in total, comprising 3 focus groups. After performing the transcription categorization and coding, comments were grouped into seven categories of usability findings. (Table 3) Changes to the mock-ups were made based on this feedback. (Figs. 3, 4).

**Table 3:** User feedback themes from the group design sessions and changes made to address the feedback

Issue	Quotes	Changes implemented
Recommendations, clinical content, and workflow did not always match what was expected by the provider	“Maybe, rather than deferring it for four weeks, maybe defer it at this visit” “Losartan usually doesn’t cause cough”	“Defer 4 weeks” to “Defer until next BP check” acknowledge reason Removed “Losartan caused cough” acknowledge reason
Providers found some elements of the design extraneous	“I, particularly, like that you can just click order, e-consult, or a referral with a click, and you don’t have to do anything else” “I don’t like his photo there. I think the whole thing is dizzying. It’s harder, for some reason, just by visual. I think the other one was easier to process and read”	Remove the order set option Remove the photo of the department head
Some providers expressed preference for the most efficient method of responding to the alert message	“I think I prefer the previous [single order version]. Seems like it’s less clicks or it takes you—I’m not sure. Once you assign this smart set, you click on a dose of lisinopril and hit the smart set, sign it. It’s gonna go into the orders section, I suppose.”	Removed the Order Set version of BPA 2A
Providers appreciated the visibility and access to important information on the BPA	“I like that the order buttons are already directly there ready to go.” “I really like the fact that you do put the female, child-bearing age warning. I think that that’s super important to remind people.”	Kept pre-selected order recommendations Kept female of childbearing age warning
Providers prefer decision support that adds clinical value and useful information at the point of care	“I think this [Minimal Information version of BPA 1A] could fall into the category of the mini warnings and alerts that I end up ignoring because it just doesn’t have useful information in it. “	Removed the minimal information version of BPA 1A
Providers preferred transparency regarding why the alert was firing and how to interact with the BPA	“I guess I could see being in the dark, and being like huh, really, why did this [BPA 1A with a link to labs and BP] fire?”	Decided against the version of BPA 1A with a link to labs and BP in favor of the version with the information included under “Why did this alert fire?”
Providers wanted information to help prevent medication errors	“I guess I would like it to take into account, if possible, if it was like, ‘Patient is not on an ACE,’ and then it would say, ‘but they had something that caused them a rash when they took irbesartan in the past’”	Added a statement regarding cross-reactivity of angioedema between ACE and ARB
Providers wanted consistency between the BPA categories	“It’s not asking for referral to renal. That, to me, seems—it’s not consistent with the previous set of BPAs”	Added both buttons to all BPAs: “Ambulatory BWH Renal E-Consult” and “Ambulatory referral to BWH Renal”

### Individual think aloud sessions

The individual think aloud sessions were conducted from May to June 2020. We recruited eight PCPs from Brigham and Women’s Hospital affiliated primary care practices. Participants had an average of 6 years of experience using the Epic EHR, and years in practice ranged from one to 40 years. All participants identified as intermediate or expert users of technology. The most common findings were that users disagreed with recommended clinical actions, requested additional clinical information, did not have consensus on informing patients of CKD and hypertension in the after-visit summary, and had concerns about the alert placement within their workflow. Some also required additional explanation of the function and behavior of acknowledge reasons. (Table 4).

**Table 4:** User feedback on the alerts during individual think-aloud sessions

Finding	Quotations
Users disagreed with recommended clinical actions	“The only thing I might change is I might start with a lower dose, and just up-titrate the losartan from there” “Need to increase that lisinopril. I've already discussed that. I frankly would go up by more than just 10.”
Users had concerns about the alert placement within their workflow and ability to navigate to other parts of the chart	“So, the problem I would have with this right now is that I haven’t talked to the patient, and it would be inappropriate for me to start a new medication without discussing with the patient”
Users requested additional clinical information	“I’d love to know what his potassium is before I start the lisinopril. If I had access to that, that would be great.” “It's also the most recent eGFR. It would be interesting to have the date here.”
Users had difficulty understanding the behavior of the Acknowledge Reasons and how they behaved in relation to the Order buttons	“Then I have to acknowledge a reason if I don’t accept it? I’m just curious how this works again.”
Users did not have consensus on informing patients about CKD and BP in their after-visit summary	“I think it would just scare patients like, “I don’t know. You didn’t tell me I had kidney disease today.”
Users had some difficulty understanding the Order buttons (3)	“I did see [the order options], but they were highlighted as do not order, so I had assumed [they were not recommended]”
Users noted that the medication order screen does not allow modification to an existing order	“It would be nice if this could program a change [of dose] instead of a new prescription so that I didn't have to discontinue.”

Based on the findings from the individual sessions, we implemented several changes (Figs. 2, 3). Users had different preferences for starting doses of medications, so we included an acknowledge reason to allow them to indicate that they intend to order a different dose. The length of time to defer the BPA was changed from 1 month to the next visit to be clearer. Finally, options to discuss with the patient and to review the chart were added to account for provider workflow since there were constraints in terms of the available trigger points in the EHR. To provide enough clinical context, we included dates for all the lab values related to diagnosis of CKD presented in the alert, as well as the most recent potassium value. Because participants expressed conflicting views on including information about CKD for the patient in the After Visit Summary, we decided to remove this. We were unable to address the issue of modifying an existing order from the alert rather than discontinuing the existing order and adding a new one since this is a constraint within Epic. In addition, we were not able to address the interaction of the order buttons and acknowledge reasons that some users had difficulty with. When the “Do Not Order” button is selected for each of the orders, an acknowledge reason is required. Once you select an acknowledge reason, all the order buttons automatically switch to “Do Not Order” even if the user previously chose to order one of them, such as a basic metabolic panel. In some cases, the provider did not notice this, thereby believing they had placed an order for a basic metabolic panel which was deleted.

## Discussion

This usability study for designing CDS for medication management of hypertension and CKD revealed the importance of: (1) incorporating the CDS with both the clinical workflow (i.e., after the BP has been checked) and clinical decision-making process, (2) providing actionable and clear recommendations, (3) including relevant contextual information, and (4) providing a simple and efficient interface. Our study showed that participation from users throughout the design process results in feedback that can be translated into user requirements and validation for design decisions.

Challenges related to the adoption of CDS such as alert fatigue and provider burden are well known. Our findings, along with those identified in other studies, highlight improvements to CDS that include removing extra clicks, allowing deferral to a later date, adding more labs and their specific dates, and making the alerts more concise and visually appealing [41, 42]. Moreover, specific challenges with providing guidance to primary care providers regarding CKD and hypertension include variation in provider thresholds for prescribing, and access to rapidly changing and often conflicting guidelines for prevention and management [50]. Our results highlight some of these challenges. Providers expressed the need for enough relevant clinical data within the alert; they also expressed a reluctance to make a prescribing decision without reviewing additional clinical data, talking to the patient or repeating measurements to ensure they consider the current context and all of the patient’s individual factors. This aligns with research that found that managing hypertension for patients with CKD is not a “one size fits all” approach but rather requires a targeted approach [43, 51]. Discovering this user need early in our design process led to design decisions that allowed the providers flexibility in deferring the alert until a later time.

In addition, some of the decisions we made addressed clinical areas without official contraindications but are common concerns. We added disclaimers about the acceptability of an increase in serum creatinine after starting certain recommended medications and a warning about teratogenicity risk for women of childbearing age.

In some cases, the provider preferences we discovered differed from previous research. For example, we previously learned that PCPs wanted a reminder to think about a referral or e-consult to nephrology and this was confirmed by primary care stakeholders during our design process [35]. However, during our testing we found that providing both an option to order a referral to renal or an e-consult with renal with each alert was not necessary. Some research suggests that there are some barriers to co-managing patients with CKD between primary care providers and nephrologists, specifically around the roles and responsibilities, which could potentially explain the reluctance to seek an e-consult [35, 36]. In addition, we found that our providers did not feel that showing a picture of the department head in the alert and having a separate link to lab data would be helpful as was found in prior research [52, 53].

We were not able to fully address some of the more frequently identified issues due to constraints of the EHR system. Ideally, we would be able to create a system that met all the user’s requirements. Providers have diverse encounter workflows. The alert firing at the start of an encounter may be a significant roadblock for some providers and lead them to ignore the alert [54]. However, providers indicated this was preferable given the EHR constraints. For those providers who rarely interact with the patient chart in the room or work closely with other clinicians, managing when and who addresses the alert is challenging. The constraints regarding the design of the order buttons and acknowledge reasons could significantly impact the user’s ability to complete the task and lead to a lot of frustration [55]. Allowing the provider to edit an existing order could improve the user’s efficiency with the system by saving several mouse clicks and add value to using the BPA. While we did have some challenges balancing the user requirements with the available solutions within the EHR, the system did offer the flexibility to turn off alerts for any individual provider. It also allowed for flexibility when presenting whether the current prescription was an ACE or an ARB for the third category of BPA that recommends initiating a diuretic on patients currently on the maximum dose of an ACE or ARB. Throughout the design process, the ability to edit the display text and add links allowed for appropriate updates following feedback on relevant data, information, and guidelines.

While the human-centered design process outlined in this study can apply to a wide range of settings, there may be some limitations to the final alert design due to the context of testing. Based on local prescription trends, we included a diuretic rather than a calcium channel blocker as the next line agent and we chose hydrochlorothiazide over chlorthalidone. These trends may vary by region or provider. The testing process was limited due to the COVID-19 outbreak, causing all sessions to be conducted remotely. As a result, we were unable to perform near-live simulation and the circumstance of being behind schedule in clinic, which impacted in-person user requirements gathering and could impact the interaction with the alert. Provider understanding of a real patient’s history may also impact their treatment plan. In addition, we were limited by our own institutions governance structure in terms of what technology solutions were allowed. Finally, the providers that agreed to participate in this testing could represent a group that would be more willing to spend the time to read through the information included in the alerts.

## Conclusion

By involving the user at multiple stages of design, we were able to refine our alerts to make them better suited for clinical decision making by providing relevant data, justification, treatment options, and acknowledge reasons. While we used this HCD framework to design CDS tailored to PCPs using Epic in the context of CKD recognition and treatment, this method can be an effective way to discover user needs for CDS regardless of the EHR or clinical scenario.

## Supplementary Information

**Additional file 1. Appendix A** Usability Test Script.

## Data Availability

The datasets used and/or analysed during the current study are available from the corresponding author on reasonable request.
