# Supplementary material for: Human-centered design of clinical decision support for management of hypertension with chronic kidney disease
Source: BMC Med Inform Decis Mak. 2022 Aug 13;22:217. doi: 10.1186/s12911-022-01962-y (PMC9375189; doi:10.1186/s12911-022-01962-y)
Supplement: Supplementary file 1 — Additional file 1. Appendix A Usability Test Script. [file 12911_2022_1962_MOESM1_ESM.pdf]

Usability Test Script

Pre-Test Instructions

Thank you for participating today. You are going to help with our research study exploring new information technology solutions for supporting chronic kidney disease management. The feedback you provide will help us to develop clinical decision support that best addresses clinicians' needs, the needs of the patient, as well as improve safety and quality.

During the session, you will walk through a few short clinical scenarios and be asked to complete a few tasks using Epic. Please speak aloud as you are using the system (talk about what you're thinking, what you're expecting to happen when you click on certain things, your reactions to content). If you are silent for a while, I may remind you to keep talking aloud. We are interested in your open and honest feedback.

My role is that of a neutral observer. I will not be able to answer questions about how do something or what something means during the scenario portion, but we can review any questions at the end.

We will be passing you control of our screen during the session so you're able to use the Epic Innovation environment. This may slow it down slightly. Also, this environment may look slightly different than your current Epic view. We will be recording the computer screen and what you are saying. The video and audio will be seen by only those people working on the research project and will be used in our analysis of the tool. **START BOTH RECORDERS**

Do you have any questions?

Pre-test Questions:

I'd like to ask you a couple of questions before we start.

1. What is your clinical role (any specialty)?
2. Approximately how long have you been practicing?
3. How long have you used EPIC?
4. What is your comfort level with technology in general? Novice, intermediate, expert

For all the scenarios today, your role will be as a Primary Care Provider in a clinic with a medical assistant who checks the blood pressure of your patients using an automated cuff. All patients will be test patients and you'll need to open an encounter for each of the scenarios. I'd like you to reach each scenario aloud and ask any questions before proceeding. Please tell me when you think you have finished addressing the task.

Scenarios/Tasks:

1. **Scenario #1 CKD Stage 3, not on ACE**

   Name: Alpha Samal Test

   A 65-year-old African American man comes to see you for a visit after a 2-year absence. You notice that he had labs checked for a pre-op six months ago and his creatinine was slightly higher than the last time you checked it at 2.2 (eGFR 35). Two years ago, his creatinine was 1.8 (eGFR 45). The patient has a previous BP of 142/80 from 6 months ago. The patient is on Amlodipine 10mg, but that is his only anti-hypertensive. Your MA has the patient sit for 5 minutes and takes his blood pressure. Their current BP is 144/78. The MA types this into the vital section of the chart and closes the chart. You enter the exam room and open the patient's encounter.

   Task: Review the alert and respond appropriately.

Additional info if prompted:

- *No diabetes.*
- *This eGFR is the same as the one you would see in "Labs" in Epic. It does not take into account race.*
- *Certain guidelines recommend Amlodipine, or any calcium channel blocker, in African American patients*

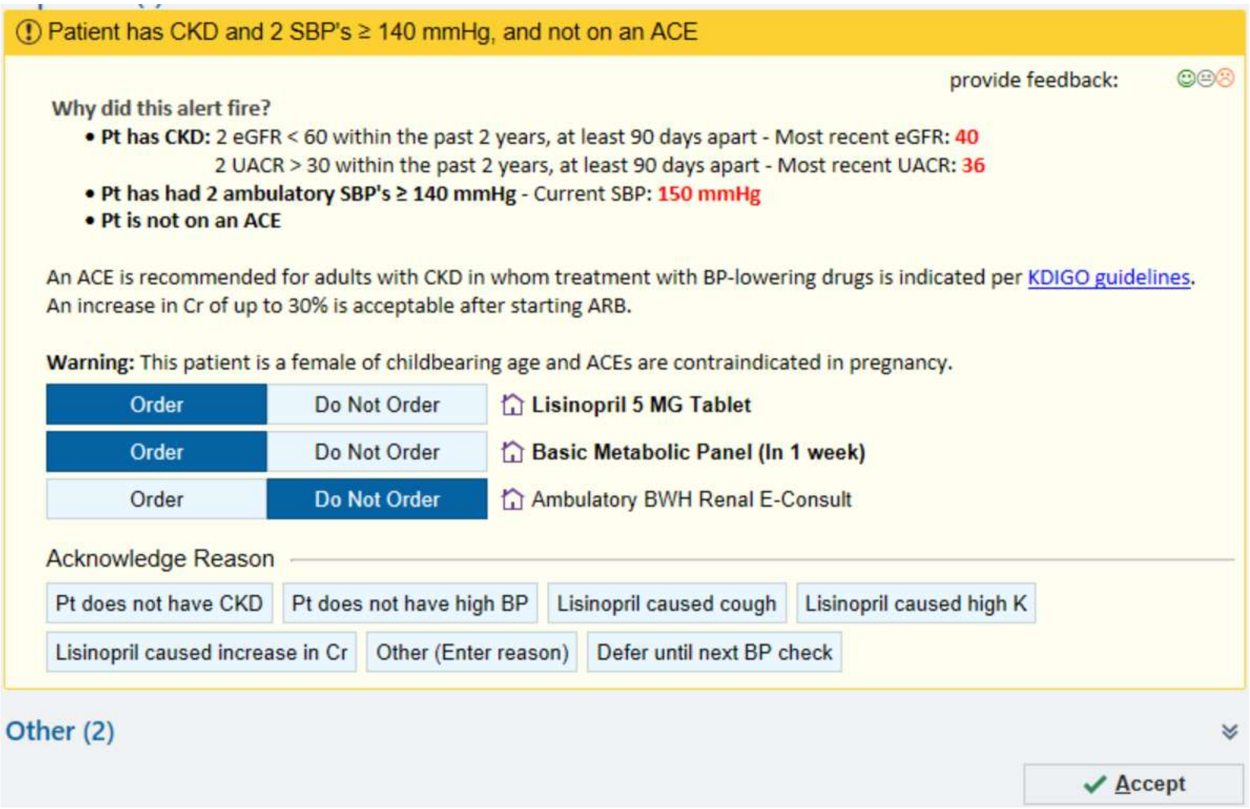

2. **Scenario #2 CKD stage 4, not on ACE or ARB with known ACE allergy**

Name: Beta Samal Test

A 48-year-old Hispanic woman comes to see you for a six month follow up for hypertension. At her last visit her creatinine was 1.6, urine albumin to creatinine ratio was 35, and eGFR was 34 mL/min per 1.73 m^2. Her blood pressure was 146/80. You started Lisinopril 10mg.

You instructed her to return to the lab before today's visit. Creatinine is 1.8, eGFR 29, and urine albumin to creatinine ratio is 46 mg/g. Prior to the visit, you reviewed her chart and noticed that she recently saw an urgent care provider who found out that she stopped taking the ACE-inhibitor because she developed a cough and noted this allergy in the chart. Your MA has the patient sit for 5 minutes and takes her blood pressure. Their current BP is 144/78. The MA types this into the vital section of the chart and closes the chart. You enter the exam room and open the patient's encounter.

Task: Review the alert and respond appropriately.

Additional info if prompted:

- *No diabetes*

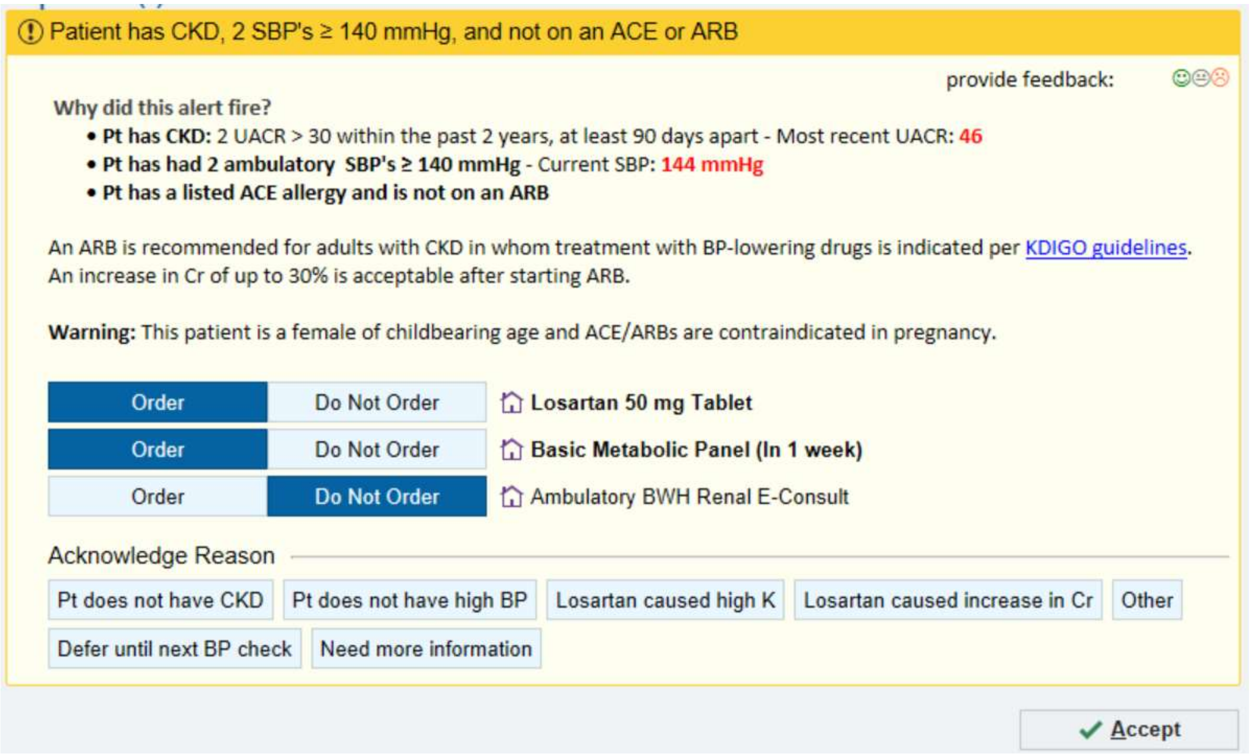

3. **Scenario #3, CKD stage 3 on low dose ACE, with added challenge of uncontrolled diabetes, prompt about referral**

Name: Charlie Samal Test

A 58-year-old white man with diabetes and hypertension comes in for a follow-up visit 5 months after you last saw him. At that time his BP was 150/82. He is on four anti-hypertensive agents—lisinopril 10mg, amlodipine 10mg, HCTZ 25mg, metoprolol XL 200mg and so you were surprised to see that his SBP was so high. In reviewing his chart, you see that his diabetes has slowly become less well-controlled over the past year, despite good adherence to metformin. He was supposed to return after 3 months to recheck his hemoglobin A1C and discuss starting a second diabetes medication, but he never made an appointment. One year ago, his eGFR was 50 and his urine albumin to creatinine ratio was 45. Five months ago, his eGFR was 40 and his urine albumin to creatinine ratio was 287. When your patient arrives, your MA has the patient sit for 5 minutes and takes his blood pressure. Their current BP is 144/78. The MA types this into the vital section of the chart and closes the chart. You enter the exam room and open the patient's encounter.

Task: Review the alert and respond appropriately.

Additional info if prompted:

- *Uncontrolled diabetes*
- *Hemoglobin A1C – 8.0*
- *Why on metoprolol? Assume indication is hypertension*
- *Patient was not able to follow the plan that was laid out. One of the priorities is diabetes.*

I noticed you did/did not choose to order a referral. Can you tell me more about that option?

Would you prefer an e-consult button alone?

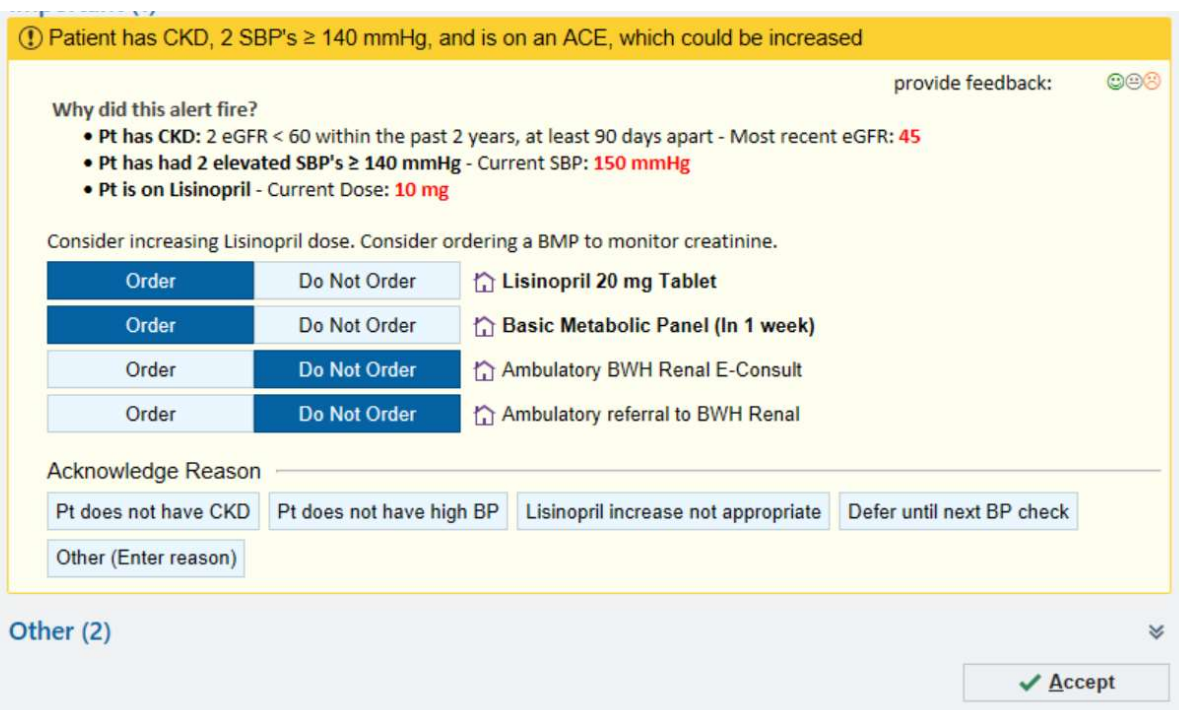

4. **Scenario #4, CKD stage 4 and on low dose ARB, with psychosocial challenges.**

   Name: Delta Samal Test

   A 62-year-old white woman returns for a 3-month follow-up visit. At her last visit her BP was 158/78 and her eGFR was 25. One year ago, her eGFR was 45. You started losartan 50 mg at that time. She has severe depression, which has been well-controlled in consultation with a psychiatrist. She has a problem with her housing and has been in close contact with social work. Today her BP is 154/82.

   Task: Review the alert and respond appropriately.

Additional info if prompted:

- *Why on ARB? Assume indication is hypertension*
- *Assume no drug interactions between anti-depressants and anti-hypertensives*

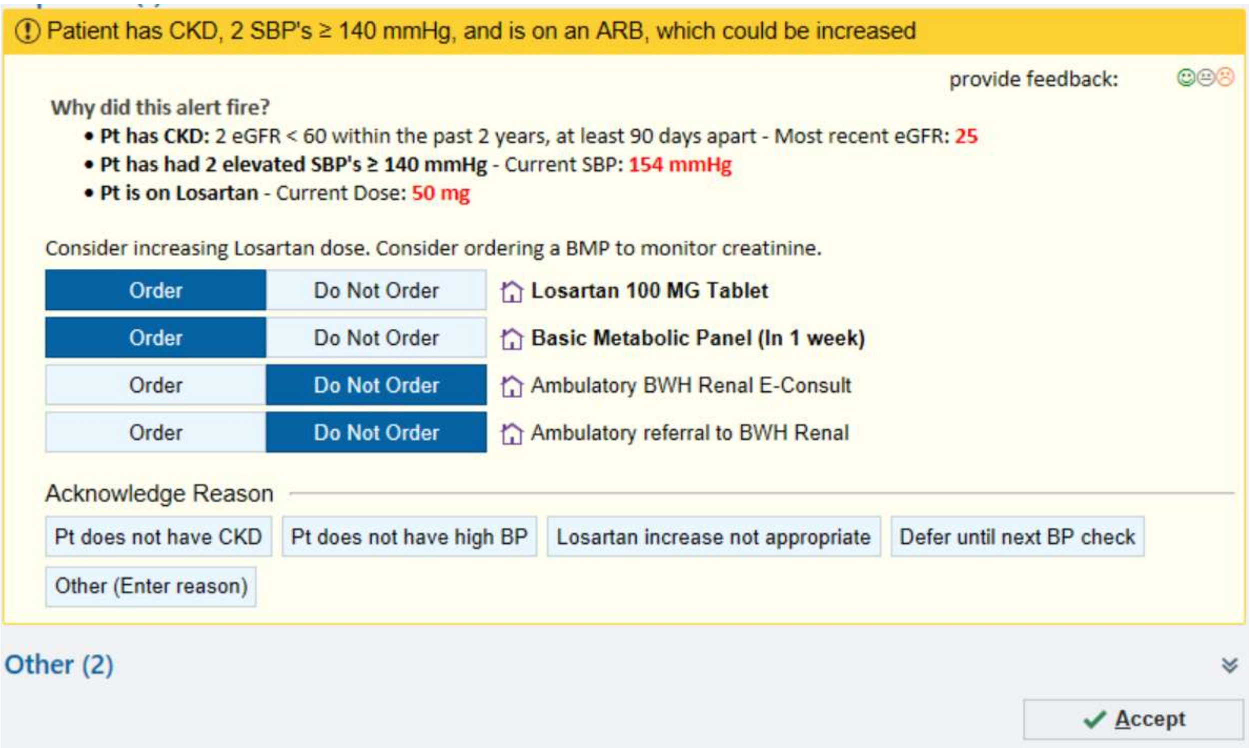

5. **Scenario #5, CKD stage 3 on max dose lisinopril, but no HCTZ, with prompt about after visit summary**

   Name: Epsilon Samal Test

   A 66-year-old African American man returns for 6-month follow-up. He is a smoker, about 5-10 cigarettes per day. One year ago, his eGFR was 50. Six months ago, his eGFR was 40. He is on lisinopril 40mg. At his last visit his BP was 158/80. Today his BP is 148/82.

   Task 1: Review the BPA and tell me what you're thinking but don't take action.

   Task 2: You want to start him on a medication to quit smoking at this visit. Tell me how you would respond to this BPA.

Task 3: You decide not to start either the HCTZ or the amlodipine today because you're starting him on the medication for smoking. Respond to the alert by clicking "Defer until next BP check".

What do you think will happen next?

Additional info if prompted:

- *Prioritize helping the patient quit smoking over anti-hypertensive changes*

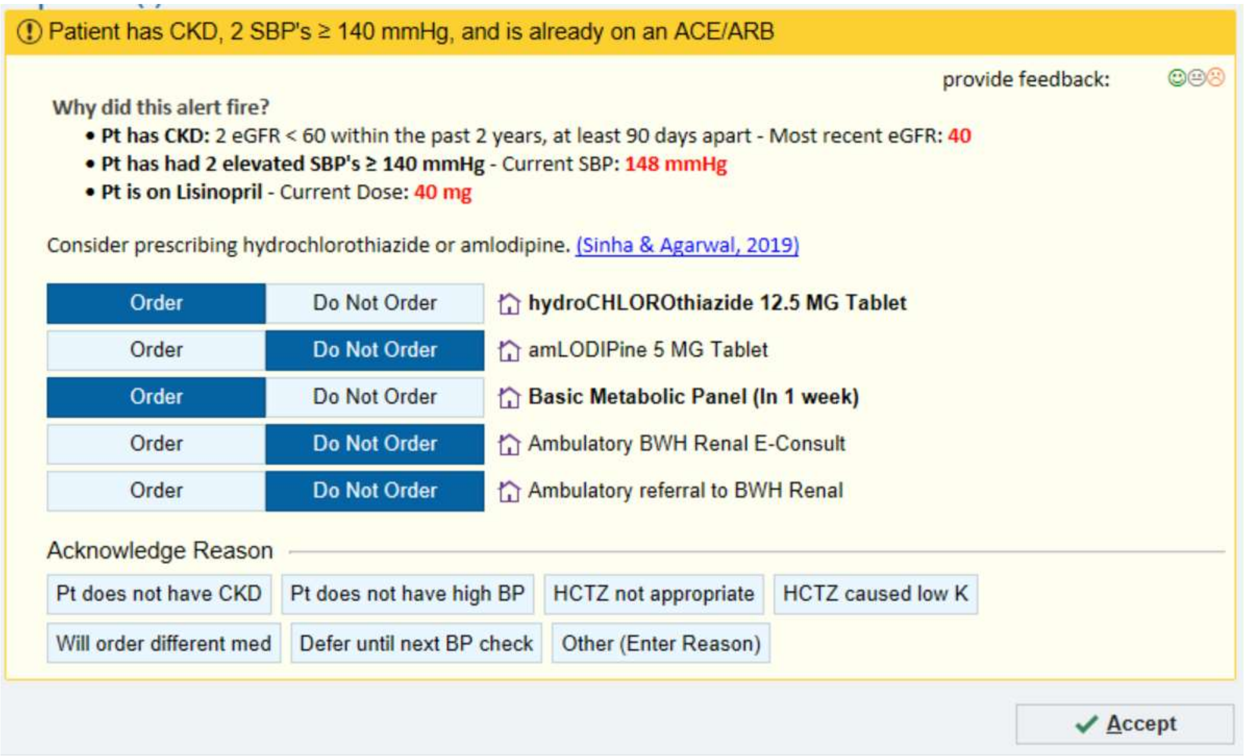

In scenario 5 we asked you to defer. Tell us more about that option. What are some other reasons you might defer? If you wanted to recheck bp before taking any of the recommendations of the BPA, would you expect it to appear again during this encounter?

Post-Test Questionnaire
System Usability Scale
© Digital Equipment Corporation, 1986.

	Strongly Disagree				Strongly Agree
1. I think that I would like to use this system frequently	○	○	○	○	○
2. I found the system unnecessarily complex.	○	○	○	○	○
3. I thought the system was easy to use.	○	○	○	○	○
4. I think that I would need the support of a technical person to be able to use this system.	○	○	○	○	○
5. I found the various functions in this system were well integrated.	○	○	○	○	○
6. I thought there was too much inconsistency in this system.	○	○	○	○	○
7. I imagine that most people would learn to use this system.	○	○	○	○	○
8. I found the system very cumbersome to use.	○	○	○	○	○
9. I felt very confident using the system.	○	○	○	○	○
10. I needed to learn a lot of things before I could get going with this system.	○	○	○	○	○

Post-Test Interview

1. What did you like about this decision support?

2. What did you dislike about this decision support?

3. What else should these alerts do or say?

4. Would you recommend your colleagues use this decision support?

5. Anything else you would like to share?
